# Detection of alpha-1 antitrypsin deficiency: the past, present and future

**DOI:** 10.1186/s13023-020-01352-5

**Published:** 2020-04-19

**Authors:** Mark Brantly, Michael Campos, Angela M. Davis, Jeanine D’Armiento, Kenneth Goodman, Kathi Hanna, Miriam O’Day, John Queenan, Robert Sandhaus, James Stoller, Charlie Strange, Jeffrey Teckman, Adam Wanner

**Affiliations:** 1 University of Florida College of Medicine, Gainesville, USA; 2 University of Miami School of Medicine, Miami, USA; 3 Grifols, Research Triangle, USA; 4 Columbia University Medical Center, New York City, USA; 5 Science and Health Writer / Editor, Seattle, USA; 6 Alpha-1 Foundation, Coral Gables, USA; 7 Queen’s University, Kingston, Canada; 8 National Jewish Health, Denver, USA; 9 Cleveland Clinic, Cleveland, USA; 10 Medical University of South Carolina, Charleston, USA; 11 Saint Louis University School of Medicine, St. Louis, USA

**Keywords:** alpha-1 antitrypsin deficiency, Alpha-1 antitrypsin, Rare disease, Detection, COPD, Chronic liver disease, Newborn screening, Electronic medical record, Direct-to-consumer testing

## Abstract

**Background:**

Most patients with alpha-1 antitrypsin deficiency remain undiagnosed and therefore do not benefit from current therapies or become eligible for research studies of new treatments under development. Improving the detection rate for AATD is therefore a high priority for the Alpha-1 Foundation. A workshop was held on June 23, 2019 in Orlando, Florida during which stakeholders from the research, pharmaceutical, and patient communities focused on the topic of alpha-1 antitrypsin deficiency detection.

**Results:**

A variety of detection strategies have been explored in the past and new approaches are emerging as technology advances. Targeted detection includes patients with chronic obstructive pulmonary disease, unexplained chronic liver disease, and family members of affected individuals. Newborn screening, electronic medical record data mining, and direct-to-consumer testing remain options for future detection strategies.

**Conclusion:**

These meeting proceedings can serve as a basis for innovative approaches to the detection of alpha-1 antitrypsin deficiency.

## Introduction

Alpha-1 antitrypsin deficiency (Alpha-1 or AATD) is an autosomal co-dominant genetic condition that can result in serious lung disease in adults and/or liver disease at any age. AATD occurs when the blood is deficient in a protein called Alpha-1 antitrypsin, or AAT. AAT is mainly produced by the liver, and its primary function is to protect the lungs from increased protease activity, in particular during episodes of inflammation caused by infection or inhaled irritants such as tobacco smoke. In most cases, a low level of AAT in the blood occurs because the misfolded AAT cannot be secreted from the liver at the normal rate. This leads to a buildup of the abnormal AAT in the hepatocytes, which can cause liver disease, and a decrease of AAT in the blood, which can predispose to lung disease.

Most patients with AATD remain undiagnosed and are therefore deprived of current specific therapies. In addition, they are not eligible for research studies involving new treatments under development. Improving the detection rate for AATD is therefore a high priority for the Alpha-1 Foundation. Indeed, detection is part of its critical mission. A variety of detection strategies have been explored in the past and some of them are still in use, However, as technology advances, there is always a need for new approaches.

With the goal of increasing AATD detection, a workshop was held following the Alpha-1 Foundation National Education Conference in Orlando, FL on June 23, 2019 to summarize the current state of the art. The workshop reviewed what has worked and what has not in past detection efforts, and introduced new detection strategies for the future. Strategies were developed to serve as a basis for future innovative approaches to the detection of AATD. This manuscript is designed to highlight the opportunities and the failures around AATD testing.

### The rationale for Alpha-1 testing

Adam Wanner, MD

University of Miami

To receive appropriate treatment people with AATD must first be identified. It is estimated that approximately 90% of patients with AATD in the United States go undiagnosed because of several obstacles. First, it is a rare disease with symptoms consistent with chronic obstructive pulmonary disease (COPD) and cryptogenic liver disease, so it is often missed. Further, part of the health care community has doubts about the therapeutic options for severe AATD and therefore does not appreciate the value of testing.

There is clear value in identifying AATD patients because specific therapy is available for AATD lung disease and because genetic counseling and family testing can identify carriers and others with the disease. Indiscriminate testing, for example, through newborn screening, direct-to-consumer tests, and mining of medical records, will yield a low rate of detection of approximately 1 in 3500. However, more targeted testing, for example, of patients diagnosed with COPD, bronchiectasis, or cryptogenic liver disease, or through testing family members of AATD patients, could yield a higher detection rate of 1 in 100. Not only is it important to identify those with AATD for reasons of clinical care, but it is also critical to increase the pool of those with confirmed diagnoses for recruitment to clinical trials of new formulations and dosing of existing drugs. Such trials are needed to develop treatments for AATD, particularly for its associated liver disease for which there are no specific therapeutic options.

Augmentation therapy with exogenous AAT is the only specific therapy for lung disease associated with AATD and its benefits are evidenced in multiple studies. A 2009 meta-analysis of five randomized controlled trials conducted by Chapman et al. supported the conclusion that augmentation can slow lung function decline in patients with AATD [1]. Patients with moderate obstruction are most likely to benefit. Further studies by Chapman et al. that measured lung density with CT at total lung capacity also provided evidence that augmentation therapy slows progression of AATD emphysema [2]. These findings endorse the value of augmentation to preserve the lung in individuals with emphysema secondary to severe AATD. The Alpha-1-Antitrypsin Registry Study Group conducted a 5-year observational study that examined decline in pulmonary function (i.e., forced expiratory volume, or FEV1) and mortality in relation to augmentation therapy and other factors [3]. It found that mortality declined in those patients receiving therapy.

New therapies on the horizon include neutrophil elastase inhibitors, gene-directed therapy, and nonspecific drugs for the lung disease. Gene silencing, small molecules, and cell-based therapies are potential new approaches to AATD liver disease. According to ClinicalTrials.gov, as of June 2019, 31 trials were actively recruiting patients to AATD trials, including: 8 trials of new formulations, applications, and dosing of existing drugs; 14 observational studies; and 9 new therapies. Phase 1 and 2 studies are focused on liver disease, cirrhosis, and COPD associated with AATD. As new therapies emerge or are on the horizon, it is critically important to increase the rate of detection of AATD to increase the pool of patients eligible to participate in trials, and to reduce the time to diagnosis by early detection. Early detection leads to improved outcomes, especially as more treatment options become available.

### Large-scale screening for alpha-1 antitrypsin deficiency: 2003–2017 results

Mark L. Brantly, MD

University of Florida

In existence since 2003, the National Detection Program for AATD is the largest targeted testing program in the United States, with more than 580,000 patient test results in its database. There are an estimated 116 million carriers of AATD worldwide and 3.4 million people who are homozygous for the disease alleles and therefore have AATD. The most common allele associated with AATD is Z, followed by S. The M allele is the normal allele. The F and I alleles are rarer and are also associated with dysfunction and COPD. The program’s testing algorithm begins with a dried blood spot obtained from an individual with COPD. An associated questionnaire collects demographic and clinical information. Genotyping is done to determine allele status. Samples that are positive for one of the dysfunctional alleles are subjected to immunogenic assays of AAT protein levels. The number of samples tested annually has rapidly increased over time, growing to more than 100,000 per year by 2017.

The program identified a cohort comprising 14.4% of 515,480 individuals with a genotype other than the normal homozygous MM. Of those, 5.9% were identified as having high-risk alleles for AATD (Z heterozygous and homozygous). The targeted testing algorithm identified 0.4% with the highest risk alleles for AATD (ZZ) compared to population prevalence estimates for ZZ of 0.021% in the general population. AAT levels by genotype showed highest levels among MM and MS samples with decreasing levels among SS, MZ, SZ, FZ, and IZ genotypes. The ZZ genotype cohort had the lowest AAT levels.

The average age at testing was lower in SZ individuals (55.7 years) and ZZ individuals (53.7 years) compared to MM types (58.4 years), suggesting that COPD brought them into medical care at an earlier age. The majority of the ZZ population was white, more than 50 years old, with no reported history of liver disease, at least one reported respiratory disease in their history, and no history of AAT augmentation therapy. ZZ individuals were more likely to be male than female. Although smoking history was similar among MZ, SZ, and ZZ genotypes. ZZ individuals were slightly more likely to have never smoked than others.

Across all genotypes pulmonologists are the most likely to test for AATD (62% for MM and 71% for ZZ), with primary care providers next most likely (30% for MM and 18% for ZZ). Allergists and hepatologists are promising groups that could be encouraged to engage in future testing efforts. The findings from the National Detection Program targeted test program support the notion that focused, simple screening methods improve the efficiency of identifying at-risk individuals for AATD and may result in the opportunity for clinical counseling, further testing, and treatment, if appropriate.

### Newborn screening

Miriam A. O’Day

Alpha-1 Foundation

Newborn screening programs are implemented by state health departments and vary by state. Despite attempts and recommendations to create a national program, none currently exists. However, legislative efforts have attempted, among other goals, to standardize national guidelines through the Newborn Screening Saves Lives Act, which established the National Advisory Committee on Heritable Disorders in Newborns and Children (ACHDNC). ACHDNC has identified 61 disorders (35 core and 26 secondary) for inclusion on its Recommended Uniform Screening Panel (RUSP). Disorders on RUSP are chosen based on evidence that supports the net benefit of screening and to inform state programs (which sometimes include screening for additional disorders not on the RUSP). Currently AATD screening is not included on RUSP or other newborn screening panels.

To be considered for RUSP a disorder has to be nominated. A nomination team is formed consisting of researchers, clinicians, advocacy organizations, and individuals. This group is referred to a specific condition review workgroup that conducts a review of the evidence, provides updates, and presents a final report to the ACHDNC. The committee then deliberates and votes based on decision criteria such as the likelihood that early detection can lead to interventions that prevent or ameliorate disease, and the technical feasibility and cost of screening.

Early detection of AATD would help patients (e.g., smoking acquisition behavior), but technical feasibility and cost of population-based screening is still a problem. If more effective treatments were available the decision to include AATD screening on RUSP would be more compelling. While the prospect of genetic sequencing in newborns could offer a diagnostic option, it also raises questions about cost, privacy, discrimination, and stigmatization, which are only partially addressed by the Genetic Information Nondiscrimination Act. In addition, better screening tools are needed, as AAT levels are not specific enough. Options for exploration include screening for circulating polymer and polymer antibodies. Feasible and cost-effective newborn screening could provide early detection with clear improvements to health, for example, influencing smoking behavior in adolescence.

### Alpha-1 coded testing and 23andMe

Charlie Strange, MD

University of South Carolina

The SERPINA1 gene that codes for AAT has more than 100 recognized variants that code for deficient or dysfunctional proteins. Many more variants occur that do not seem to change protein structure or function. However, most AATD patients have deficiency-associated variants that occur with point mutations at one of two sites of the allele. The first of these, rs28929474, is commonly called the protease inhibitor (PI) “Z” mutation (PI*Z, or Z); the second, rs17580, is commonly called the protease inhibitor (PI) “S” mutation (PI*S, or S). Because both of these allele variants are common and found in 2–3% of the U.S. population, targeted testing for these is commonly performed in lieu of gene sequencing. This effort to make complex genetics easy to understand has provided a robust platform for the AATD patient community to engage with family testing initiatives.

Staring in 2001, the Alpha-1 Coded Testing (ACT) Study was based at the Medical University of South Carolina in Charleston as a free and confidential research study. After obtaining online informed consent, a research questionnaire and a finger-stick blood spot card are mailed to the individual’s home. The completed test is then mailed back to the University of Florida and results are returned confidentially to the patient. Support through a genetic counseling service completes the program. ACT provides a way for those at risk for AATD, including family members of already diagnosed patients, to learn their genotype.

This type of targeted testing of families produces a high level of uptake (81%) and a high AATD detection rate. The questionnaire collects data on the perceived benefits and risk of testing, quality of life and family functioning, health locus of control, secondhand smoke, and comorbidities. Test results for the first 32,210 participants show that 4.8% of the study population have clinically significant genotypes (PI*SZ, PI*SNull, PI*ZNull, PI*ZZ). Importantly, the opportunity for families to have access to confidential and free AAT testing allows participants to decide about testing on their own terms. Research into the ACT platform has shown that patients value testing [4] including the parents of minors [5], engage with their physicians around test results, and change health behaviors after testing such a smoking [6]. Barriers to testing include fear of finger-sticks with a lancet at home [7], which could be offset by buccal swab collection of DNA.

A new ACT question asks about the reasons for testing, with one choice being “I learned about testing through a large panel genetic laboratory (e.g., 23andMe, Ancestry, Promethease, or other).” Numerous direct-to-consumer (DTC) genetic testing options are available and consumers currently have many companies to choose from for large panel genetic testing. Strange and colleagues pulled out for analysis the 135 individuals who responded that they came to ACT because of results received through whole genome sequencing conducted by these commercial laboratories. They were overwhelmingly female (primary health seekers) (77%), Caucasian (94.8%), and with an average age of 41.5 years. Sixty-two percent had read about AATD on the Internet. Of significance, those who had undergone whole genome sequencing were less likely to report chronic lung disease than all those tested (20.7% versus 34.9%) and less likely to report chronic liver disease (5.3% versus 12%). Among those first tested somewhere else, the Alpha-1 variants causing severe deficiency were found in 7.5% of the sample.

This group of 135 reported that they sought ACT testing because they had a family member with the disease, a partner with the disease, or at the urging of someone they knew with AATD. Other reasons included encouragement by a physician or respiratory therapist, or because of information obtained through the Alpha-1 Foundation website, brochures, videos, or genetic counseling services. 23andMe offers testing for Alpha-1 variants (as approved by the Food and Drug Administration); however, most DTC companies do not test for Alpha-1 and even 23andMe only tests for the major alleles (S and Z). If a person tests positive for one or more variant alleles they are provided information about associated risks of lung or liver disease, ways to mitigate risks (e.g., avoiding smoking or other occupational exposures), and where to seek additional information. Consumers can opt into research use of their raw data.

In sum, next generation sequencing, like most genetic testing, is driven by female influences on health seeking behavior and family testing will continue to be an important strategy for targeted testing. Further, health care test seeking is heavily influenced by what is available on the Internet. As such, Alpha-1 Foundation Internet resources and genetic counseling services are used frequently by this population.

### Detecting rare disease in electronic medical records: a proof-of-concept study using symptoms associated with Mucopolysaccharidosis

John A. Queenan, PhD

Queen’s University

The Canadian Primary Care Sentinel Surveillance Network (CPCSSN), founded in 2008, is a national effort involving a partnership of 13 Canadian universities [8]. The data are extracted quarterly, anonymized, cleaned, and coded, then mapped to a common database structure. Structured data sets include provider profile, patient sociodemographics, disease/health condition, encounters, risk factors, examinations, medications laboratory tests, referrals, and procedures. The data are used for chronic disease surveillance, research, quality improvement, and panel management. CPCSSN is representative of a population of patients under the care of Canadian primary care practitioners [9]. Patients can opt out of the network consistent with research ethics board approvals, and all data are de-identified and stored in a highly secure facility with all personnel held to strict privacy and confidentiality requirements [8]. An ethical framework guides working with industry partners and individual networks can and do opt out of industry-sponsored research.

Recently, CPCSSN conducted a proof-of-concept study to determine whether a rare disease could be detected through analysis of routinely collected electronic medical record (EMR) data [10]. The exemplar condition chosen was mucopolysaccharidosis type II (MPS II), a rare and potentially life-threatening multi-system disease in males [11]. MPS II needs to be identified as early as possible due to its progressive course and to implement enzyme replacement therapy in a timely manner. It is usually diagnosed from about 18 months to 4.5 years of age and displays a range of severity for which individual symptoms are not specific [12, 13]. The primary objective of the study was to estimate the prevalence of MPS II in the Canadian primary care population. A second objective was to establish the feasibility of employing a method for identifying a high-risk population, that has not yet been diagnosed by analyzing the database by symptom categories and groupings [10].

This was a retrospective cohort study restricted to males between the ages of birth and 17 years (*n* = 152,821). The team previously developed a Naïve Bayes classification (NBC) algorithm utilizing the clinical diagnosis and symptoms of patients contained within their de-identified and unstructured records [10]. Sixty-seven patients were identified with symptoms that indicated an increased risk of having an undiagnosed case of MPS II. Most of these patients were under the age of 4 years. The next step would be to confirm the diagnosis in the 67 patients identified; however, the team would have to obtain permission to re-identify these individuals and careful consideration must be given to a design that would minimize harm (anxiety and concern of those patients and families who are false positive) and the need to clearly identify the benefits to those who had previously been undiagnosed.

The study team concluded that it is possible to identify a candidate list of patients who may have undiagnosed, rare disease in an EMR database using clusters of suspicious symptoms. This technique may be applicable to AATD; one could consider identifying a cohort of COPD patients who are free of traditional COPD-related risk factors but have the symptoms associated with COPD. Individuals who are identified could then be contacted and invited into a study.

### Medical record-based reminder in the Miami VA medical center

Michael Campos, MD

University of Miami Miller School of Medicine

Testing COPD patients is one of the main objectives of targeted detection of AATD. It is known that two-thirds of patients with COPD are first encountered and treated by nonspecialists, in particular, primary care physicians [14]. However, COPD is a condition that remains highly underdiagnosed in the United States [15]. In the National Health and Nutrition Examination Survey (NHANES) it was found that greater than 70% of subjects with confirmed airflow obstruction were not clinically diagnosed [16]. Thus, it is imperative to improve COPD detection in order to increase AATD detection. Toward this goal, the Miami Veterans Administration Medical Center (VAMC) conducted a study of the utility of using EMR reminders to increase COPD detection and AATD targeted screening in a primary care population. An electronic clinical reminder was created in the EMR (called Computerized Patient Record System or CPRS) with an algorithm prompting assessment of COPD risk (the COPD Population Screener or COPD-PS) [17], followed by spirometry ordering for subjects with high scores, an interpretation dialog, and prompting screening of AAT by ordering AAT levels when airflow obstruction (i.e., COPD) was confirmed in high-risk patients.

Of 9497 veterans identified as at risk, 8458 (89%) underwent the COPD-PS questionnaire and 585 (6.1%) did not because of an existing COPD diagnosis. Excluding subjects younger than 40 years old, 13.3% of the primary care population was found to be at high risk for COPD. There was an overall increase in the ordering of spirometry in both high-risk for COPD (83% versus 24.4%) and known COPD (84.2% versus 58%) groups, which resulted in less over- and under-diagnosis of COPD. A new diagnosis of COPD was made in 43% of the high-risk patients and a correction of an over-diagnosis (misdiagnosis) of COPD was made in 33% of the so-called COPD patients. The reminder increased AATD screening 4.2 times in these subjects with newly confirmed COPD, with 78.4% tested with AAT levels.

In conclusion, an EMR reminder, at least in a “closed” health care system such as the VA, is an effective tool for COPD case finding, decreasing COPD over diagnosis, and increasing AAT testing (which is less ideal than ordering genotyping but more easily ordered and understood by clinicians). The reminder system was well received by clinicians and patients, with only a low percentage (5%) refusing to undergo screening. It provides proof that “automatic” AATD testing is possible when tagged along with a condition that practitioners consider more common and important. Going forward, a better COPD screener is needed with improved sensitivity and specificity.

### Detecting alpha-1 antitrypsin deficiency through pulmonary function testing and provider education

James K. Stoller, MD, MS

Cleveland Clinic

Under-recognition of AATD is common and persists currently. A 1989 study by Silverman et al. found that 96% of patients go undiagnosed either because they have no signs or symptoms or because they are symptomatic but have been misdiagnosed [18]. Luisetti and Seersholm found that across seven European countries and Canada, on average, only 0.35% of expected cases of AATD were actually diagnosed [19]. Detection can be advanced by providing advice with test results to ordering physicians and by educating and activating respiratory therapists.

In addition to under diagnosis, there is a long diagnostic delay between the time symptoms first appear and initial diagnosis The mean interval between first lung symptom and first diagnosis was found to be 7.2 years on average in a study by Stoller et al. [20] In the same study, 43.7% of patients reported that at least three doctors were seen before the diagnosis was made; 25.1% reported that the first doctor they saw made the diagnosis. Significantly, this delay has not improved [21]. There has also been no significant decrease in the number of physicians seen before initial diagnosis over time [22]. These delays are critical because they are associated with worsened clinical status at the time of diagnosis. Tejwani et al. found that for each additional year of diagnostic delay, the St. George’s Respiratory Questionnaire (SGRQ) score increased 1.7 points on average, indicating negative impact on overall health because of obstructive airways disease [23]. Similarly, a significant association between higher COPD Assessment Test (CAT) Score and diagnostic delay was seen, as was a trend toward lower FEV1 and longer diagnostic delay.

These findings underscore the need to increase testing and reduce the diagnostic delay. Several studies have reported on successful strategies to improve diagnoses. Rahaghi et al. found that annotating pulmonary function test reports in patients with obstruction prompts physicians to test for AATD, increasing testing from 6 to 13% [24]. Even so, while annotating the written report was associated with an increased prevalence of testing for AATD in at-risk populations, testing for AATD was still conducted in a small minority of eligible patients and no individuals with severe deficiency of AAT were newly detected, not unexpected because of the small numbers involved.

In a prospective study Jain et al. assessed whether an electronic alert in the EMR could enhance testing for and detection of AATD. The alert suggested testing for AATD whenever pulmonary function tests results showed airflow obstruction [25]. Providers then opted in to order a test for AATD. After implementing the alert system, 15.1% of subjects were tested for AAT deficiency versus 4.7% before. However, there was no significant increase in detecting AAT deficient individuals or in the rate of detecting ZZ individuals.

Choudry et al. used the EMR to identify COPD patients for targeted detection of AATD. Of 1247 patients identified, 8.7% showed up for testing [26]. Of these, one person was ZZ and the vast majority (93%) were MM. This study demonstrates the value but also the limits of an opt-in strategy to encourage providers to test more often. In contrast the effort by Campos (described above) is an opt-out strategy, which may be more promising.

A small minority of people with COPD see a pulmonologist, which means that if this population is to be targeted for AATD detection, other health care professionals will need to be enlisted. However, there is a knowledge gap regarding AATD among respiratory therapists and internal medicine trainees [27]. Regrettably, conventional provider continuing education alone has had little impact on the rate of under detection of AATD over nearly three decades [28].

In 2014, Stoller et al. published results of an online training program for respiratory therapists created by the American Association for Respiratory Care and the Alpha-1 Foundation [29]. Between July 2012 and June 2013, 378 therapists took the online program and 326 patients reported that they were referred for testing by a respiratory therapist. Thirty-four percent of these referrals were by the newly trained therapists. Of 62 test blood kits returned by these 111 referred patients, two were from patients identified as having severe AATD (ZZ) and one from a SZ patient. This proof of concept demonstrated that educating respiratory therapists about AATD was associated with referral of patients for testing and high rates of detection of individuals with disease. Another study found that respiratory therapists at the point-of-care (i.e., pulmonary function testing laboratories) can increase testing for AATD by identifying patients with airflow obstruction [30]. Other similar studies are underway.

As illustrated by these studies, strategies to enhance detection may include empowering allied health providers, particularly respiratory therapists, to detect AATD through judicious use of the EMR, training, and linking advice to test for AATD to pulmonary function tests, whether written or EMR-based.

### Direct-to-consumer genetic testing

Kenneth W. Goodman, PhD

University of Miami Miller School of Medicine

Direct-to-consumer testing raises questions about quality and accuracy, who should be tested, and for what purposes. Although commercial testing is technologically sound and required to meet certain quality standards, such as those required under the Clinical Laboratory Improvement Amendments of 1988 (CLIA), consumers are cautioned that results are not necessarily conclusive or predictive.

Some companies market their products as entertainment as well as science, but, more importantly, we should be asking about clinical validity, the use of such tests in children, commodification of these data, potential effects on a person’s access to insurance, and whether people are providing valid consent to the clinical and research use of their genetic information. Are consumers getting sufficient information about the possible consequences of genetic testing in deciding whether to purchase a test? These questions require that we become smarter about how we employ and deploy this technology. The delivery and interpretation of probabilistic health data requires accompanying disease surveillance, adequate oversight, clinical rigor, and robust referrals and care. Further, the technology should be patent-centric and not just profitable.

Appropriate use of these tests can be achieved through improved public education and science literacy as well as professional development of health care providers, who should be reliable and credible sources of information for the consumer trying to interpret test results. Rules and regulations should require robust security for protecting genetic data, penalizing inappropriate use, fostering professional standards, and emphasizing functions and results. Governance and oversight of direct-to-consumer genetic tests should aim to balance values and data, identify best practices, update the focus of research ethics reviews to accommodate new technologies and big data, and ensure there is a consultation capacity for risk communication to assist individuals in making decisions under conditions of uncertainty.

### Expanding the identification of deficient alleles

Angela M. Davis, MD

Grifols

Grifols has developed a new genetic assay for AATD, the Progenika A1AT genotyping test, approved by FDA in 2017. The assay is a polymerase chain reaction hybridization-based in vitro diagnostic test that uses the Luminex® 200™ instrument with xPONENT® software to simultaneously identify allelic variants. It performs simultaneous typing of the SERPINA1 gene for the 14 most prevalent mutations associated with AATD. The study that supported the regulatory submission compared the in vitro diagnostic test with whole blood to bidirectional Sanger sequencing. A total of 116 DNA samples, representing as many variants, were interrogated by the assay and the results showed 100% concordance between these two methods. The current Grifols supported testing process focuses on the F, I, S, and Z alleles; however, the new process will assess 14 alleles.

The company will be rolling out a quick and easy-to-use buccal swab kit for oral sample collection. No drying time is required for this method and DNA integrity is maintained at ambient temperature for transportation via regular mail, with the sample remaining stable for 2 months. The kit has a shelf life of 2 years.

This buccal swab kit with the new assay has been piloted in Spain. Through early 2019, 2899 tests have been processed through the Progenika labs in Bilbao, Spain. The average processing time from sample collection to results is 7.1 working days. At this point, 24 patients were identified as carrying the ZZ alleles and 49 were SZ. In market research conducted after experience with the buccal swab test, 8 out of 10 pulmonologists in Spain preferred the buccal test over the dry spot test. This test provides an important targeted screening opportunity, acknowledging that some patients will need confirmatory testing of genotype and AAT level. There will be reference laboratories in the United States once the buccal kit is available.

### Adolescent testing

Jeanine D’Armiento, MD, PhD

Columbia University

Adolescent medicine (also called herbiatrics) is a medical subspecialty that focuses on care of patients who are in the adolescent period of development. This period begins at puberty and lasts until growth has stopped. Preventive medicine is the key to adolescent medicine, primarily focused on pregnancy, substance abuse (including smoking), mental health, and sexually transmitted diseases [31]. The field is growing with the number of adolescent centers increasing across the country. These centers focus on risky behaviors but also are beginning to offer preventive medicine in the form of lipid and anemia screening, screening for drug and alcohol abuse and depression, testing for sexually transmitted disease and HIV, pregnancy testing, and providing safety advice [32].

Although adolescents resist being told what to do, if they are at increased risk for a health condition because of a predisposition, they should have that knowledge so they can make informed decisions regarding lifestyle factors that can escalate that risk. Genetic testing is, however, not as straightforward since in contrast to immunization policies, which either require or recommend vaccination as preventive measures, genetic testing can label the adolescent with a disease, which has numerous implications. It is nonetheless beneficial for adolescents to be informed of their potential risks with regard to AATD. Clearly ZZ patients would benefit from knowledge about the disease, but recent evidence also demonstrates that AATD heterozygotes should also be informed of their status as it is known that cigarette smoke is a risk factor for AATD in both ZZ and MZ patients [33] and both ZZ and MZ patients can develop liver disease [34]. Adolescent risky behavior, such as smoking, can increase the chance of developing symptomatic AATD. Knowledge of carrier status may assist health care providers in emphasizing the need to avoid risky behaviors and encourage adolescents to take greater precautions. Further, an adolescent might modify his or her occupational choice based on awareness of AATD status (e.g., avoid hazardous occupational exposures in career choices).

Identifying adolescents who have AATD or are who are carriers bears risks as well as potential benefits. It creates a pre-existing condition that might be the basis of denial for life or disability insurance. Minors also cannot provide true informed consent but rather assent, with their parents’ consent, which raises the issue of whether they would have consented to testing once they reach the age of majority. It was suggested that the Alpha-1 Foundation enter into discussions with professional medical societies focused on pediatric and adolescent health in addition to consulting with the Alpha-1 community concerning possible strategies for or approaches to adolescent testing.

### Liver disease based detection

Jeffrey Teckman, MD

St. Louis University School of Medicine

The liver disease associated with AATD poses numerous challenges with regard to detection. An added complexity is that AATD affects the liver differently at different ages. In infants it appears as cholestatic hepatitis while in the pediatric population it manifests as chronic liver disease. Because there is no liver treatment for AATD, it is not a candidate for newborn screening at this time. Further, a testing method would have to be developed if AATD-associated liver disease was included in newborn screening. There is evidence that serum A1AT polymer is detectable at a young age and it is easily identified in a dried blood spot, suggesting it might be of value as a newborn screening tool.

Symptomatic ZZ infants and children are diagnosed efficiently and quickly because their symptoms are unique (e.g., regarding growth, nutrition, and development) and pediatricians are more familiar with rare diseases than other health care specialists. Children and teens with liver symptoms, signs, or abnormal tests are typically tested for AATD if acute infections, toxins, or congenital anomalies are ruled out.

In contrast, symptomatic ZZ adults are not efficiently diagnosed, and are often mistaken for alcohol injury or nonalcoholic fatty liver disease (NASH). The early to middle adult is likely to present with lung disease but rarely with a new liver diagnosis, but older adults are more likely to present with increasing liver disease, chronic hepatitis, and cirrhosis. Specific serum testing for AATD should be consistently performed in adult work ups for liver disease as liver biopsy cannot be relied on for diagnosis, either positively or negatively. A liver biopsy from a ZZ adult is easily mistaken for NASH unless specialized tests are done. Liver disease has a wide spectrum of signs and symptoms and the liver has many functions that do not fail consistently or equally. Test results vary widely in usefulness and sensitivity in a given individual. An effective liver treatment will accelerate the demand for adult diagnosis.

To improve diagnosis in adults, several steps can be taken: raise awareness that fatty liver can be caused by ZZ; make phenotype an early adult second tier test, maybe as a follow-up to a diagnosis of NASH in all cases; and raise awareness that a liver ultrasound reading of “fatty liver,” which is a common report, is not accurate, not specific, and might be edema or cirrhosis from any cause.

Continuity of care is essential once a diagnosis is made. Patients need both lung and liver evaluation by an experienced physician, including ultrasound of the liver and spleen. They need a liver focused history and physical examination, basic labs, and a noninvasive measure of fibrosis. Further, these patients should be monitored annually for liver involvement, with more frequent visits if there is evidence of progressively worse excretory function, synthetic function, or fibrosis. Early detection also provides an opportunity for lifestyle modifications that can improve overall outcomes for AATD patients.

### Assessment of the past, present, and future

Speaker Panel

The presentations of this workshop described the past and the present, with some suggestions for the future state of AATD testing. In a concluding session, attendees discussed the testing approaches presented and their implications for future approaches. Most importantly, early detection has been shown to result in better health outcomes, not only as a result of treatment but also because at-risk individuals have the opportunity to make lifestyle choices to lower their risk. Broad population-based screening programs are likely to be expensive and yield a low rate of detection. As such, targeted testing approaches are like to be more productive.

Much of the discussion focused on the COPD population. One discussant noted that the term “COPD” was developed as a way of bringing public health attention to chronic bronchitis and emphysema, but it has also resulted in less clarity and insufficient categorization of phenotypes, including AATD. In identifying new testing approaches and developing targeted therapies, it will be important to “unbundle” COPD so that AATD testing and treatment has a clearer path forward. Asthma serves as an example of the value of further subtyping a disease for targeted treatment approaches. Developing an endotype of AATD as a subtype of COPD is important.

Because the COPD population is large and there are targeted marketing campaigns for COPD treatments (and therefore increased awareness), it is also a logical starting point for more aggressive AATD testing programs. As presented during the workshop, EMR reminders and links to, for example, pulmonary function test results, are effective means for increasing testing for those diagnosed or with symptoms of COPD. Likewise, education programs for health care providers treating people with COPD, such as respiratory therapists, have been shown to improve uptake of AATD testing. Campaigns to encourage people with COPD to request Alpha-1 testing could be considered.

Individuals increasingly have the opportunity to purchase direct-to-consumer tests and learn about AATD risk on the Internet. While these trends provide opportunities for expanding the number of people being diagnosed, caution is also in order to ensure test results are administered, reported, and interpreted appropriately. Since women tend to be the health locus of control and more likely to seek health information on the Internet, appeals could be targeted to women in terms of encouraging family members to seek testing.

As new testing platforms become available, such as the Grifols buccal swab, the cost of testing will decrease and its ease will increase. As these new tools are introduced, it will be important to track who is ordering them, as that will provide needed information for future testing and marketing strategies.

Finally, as more research studies sequence whole exomes or genomes, the Alpha-1 community has to consider how to engage in discussions with professional and policy groups about the reporting of an incidental findings of an AATD allele in some research subjects. Currently, AATD does not rise to the level of recommended conditions for which incidental findings should be reported, which poses a missed opportunity for individuals in terms of treatment and family testing.

## Conclusions

Most patients with AATD remain undiagnosed and do not benefit from specific therapy. A major reason for the under-diagnosis is that while a rare disease, its clinical manifestations are consistent with common conditions such as COPD) and chronic liver disease, so the diagnosis is often missed. In an attempt to increase the detection rate, a variety of strategies have been explored in the past and new approaches are emerging as technology advances. These include targeted detection in patients with COPD or unexplained chronic liver disease, testing of family members of affected individuals, newborn screening, electronic medical record data mining, and direct-to-consumer testing. Some of these strategies are already included in clinical practice guidelines while others remain to be validated and implemented. These meeting proceedings can serve as a basis for innovative approaches to the detection of AATD.

## Data Availability

No original data were included in the manuscript. As a meeting proceedings, the manuscript is a summary of meeting presentations with relevant references included in the manuscript.
